# Deciphering the role of lncRNA-mediated ceRNA network in disuse osteoporosis: insights from bone marrow mesenchymal stem cells under simulated microgravity

**DOI:** 10.3389/fmed.2025.1444165

**Published:** 2025-04-03

**Authors:** Wuzeng Wei, Zhongli Zhang, Bing Li, Zhe Fu, Jun Liu

**Affiliations:** ^1^Department of Orthopaedics, Tianjin Hospital, Tianjin University, Tianjin, China; ^2^Clinical College of Orthopedics, Tianjin Medical University, Tianjin, China

**Keywords:** disuse osteoporosis, lncRNA, WGCNA, competing endogenous RNAs, BMSCs, CMAP

## Abstract

**Background:**

Disuse osteoporosis (DOP) poses a significant health risk during extended space missions. Although the importance of long non-coding RNA (lncRNA) in bone marrow mesenchymal stem cells (BMSCs) and orthopedic diseases is recognized, the precise mechanism by which lncRNAs contribute to DOP remains elusive. This research aims to elucidate the potential regulatory role of lncRNAs in DOP.

**Methods:**

Sequencing data were obtained from Gene Expression Omnibus (GEO) datasets, including coding and non-coding RNAs. Positive co-expression pairs of lncRNA-mRNA were identified using weighted gene co-expression network analysis, while miRNA-mRNA expression pairs were derived from the prediction database. A mRNA-miRNA-lncRNA network was established according to the shared mRNA. Functional enrichment analysis was conducted for the shared mRNAs using genome ontology and KEGG pathways. Hub genes were identified through protein-protein interaction analysis, and connectivity map analysis was employed to identify potential therapeutic agents for DOP.

**Results:**

Integration of 74 lncRNAs, 19 miRNAs, and 200 mRNAs yielded a comprehensive mRNA-miRNA-lncRNA network. Enrichment analysis highlighted endoplasmic reticulum stress and extracellular matrix (ECM) pathways as significant in the ceRNA network. PPI analysis revealed three hub genes (COL4A1, LAMC1, and LAMA4) and identified five lncRNA-miRNA-hub gene regulatory axes. Furthermore, three potential therapeutic compounds (SB-216763, oxymetholone, and flubendazole) for DOP were identified.

**Conclusion:**

This study sheds light on the involvement of lncRNAs in the pathogenesis and treatment of DOP through the construction of a ceRNA network, linking protein-coding mRNA functions with non-coding RNAs.

## 1 Introduction

While space exploration captivates our imagination, it also presents a spectrum of short- and long-term health challenges for astronauts (1). One of the most critical concerns is disuse osteoporosis (DOP), a condition characterized by accelerated bone loss due to mechanical unloading in microgravity environments (2, 3). Astronauts can experience a bone mass reduction of up to 2% per month during spaceflight, equivalent to more than a year’s worth of bone loss in postmenopausal women (4). Disuse osteoporosis (DOP), arising from skeletal mechanical unloading, underscores the importance of investigating bone health under microgravity conditions (5).

Bone homeostasis depends on the balance between bone resorption by osteoclasts and bone formation by osteoblasts, processes critically regulated by bone marrow mesenchymal stem cells (BMSCs) (6, 7), which can differentiate into various cell types, including osteocytes, osteoblasts, chondrocytes, adipocytes, and smooth muscle cells (8). Strict regulation of BMSC differentiation is pivotal for maintaining bone homeostasis, as even minor disruptions can lead to orthopedic diseases such as osteoporosis (9). Although previous studies have explored the involvement of protein-coding genes in BMSC differentiation, increasing evidence suggests that non-coding RNAs, including long non-coding RNAs (lncRNAs) and microRNAs (miRNAs), also play pivotal regulatory roles in this process.

Long non-coding RNA (lncRNA), a subgroup of non-coding RNAs exceeding 200 nucleotides in length, have emerged as key players in diverse cellular processes such as cell differentiation, apoptosis, gene regulation, and cancer progression (10). Recent research has implicated lncRNAs in the regulation of osteogenic differentiation in BMSCs, with aberrant expression potentially disrupting bone homeostasis (11). For instance, decreased expression of lncRNA-H19, induced by mechanical unloading, has been linked to DOP development through Wnt signaling inhibition via increased Dkk4 expression. MiRNAs are known to modulate bone metabolism under mechanical stress and are associated with disuse-induced osteopenia or osteoporosis (12). lncRNAs regulate gene expression through various mechanisms, including acting as ceRNAs that sequester miRNAs from their target mRNAs. This competitive mechanism forms lncRNA-miRNA-mRNA networks, which are involved in many physiological and pathological processes (13). While their importance is recognized, few studies have explored the roles of competing endogenous RNAs (ceRNA) networks in DOP, particularly under microgravity conditions. Understanding these networks could provide valuable insights into the molecular mechanisms of DOP and reveal novel therapeutic targets.

This study addresses a critical gap in the understanding of DOP by focusing on the underexplored role of non-coding RNAs (ncRNAs), particularly lncRNAs, in regulating bone homeostasis under microgravity conditions. While previous research has primarily focused on protein-coding genes, our work highlights the complex interplay within lncRNA-miRNA-mRNA networks and their involvement in key processes, such as endoplasmic reticulum (ER) stress and extracellular matrix (ECM) remodeling. By constructing a comprehensive competitive endogenous RNA (ceRNA) network through multi-omics integration, this study not only advances the molecular understanding of DOP but also identifies novel therapeutic targets.

Furthermore, the use of weighted gene co-expression network analysis (WGCNA) allowed us to systematically identify key regulatory axes, while Connectivity Map (CMap) analysis revealed bioactive compounds with strong translational potential, including SB-216763, oxymetholone, and flubendazole. These findings provide a foundation for developing targeted therapeutic strategies for DOP, bridging the gap between bioinformatics insights and practical applications. By filling these research gaps, our study paves the way for more effective prevention and treatment of DOP, offering significant benefits for astronauts and patients with osteoporosis caused by prolonged bed rest.

## 2 Materials and methods

**FIGURE 1:**
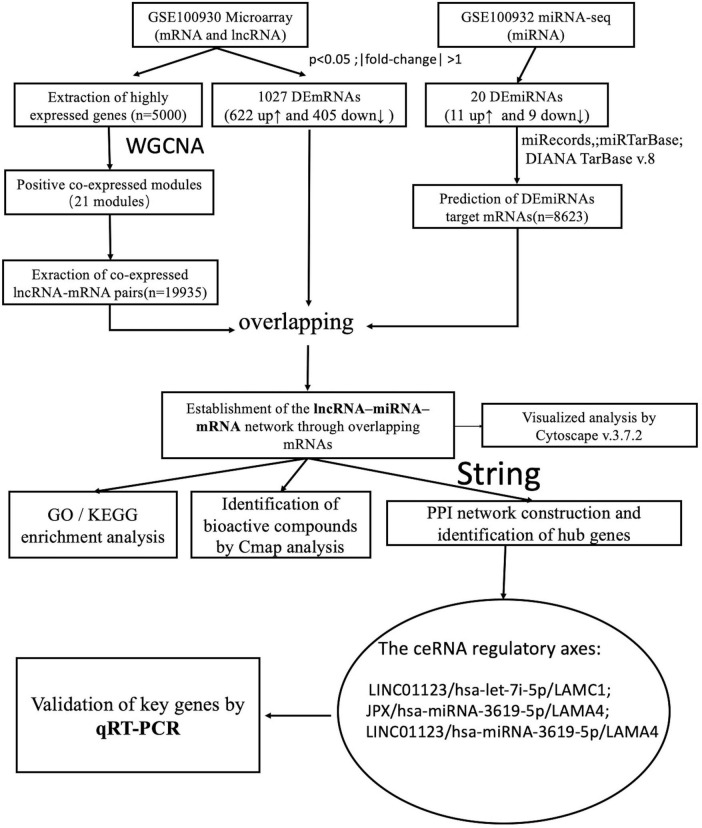
Flow chart of the analysis in this study.

A summary of the analysis procedure employed in this study is presented in Figure 1, while fundamental details regarding the two microarray datasets are outlined in Table 1.

**TABLE 1:** Basic information on the two datasets from Gene Expression Omnibus (GEO).

GEO datasets	Year	Country	Platform	Sample	N	Detected RNA type
GSE100930	2019	Italy	GPL6244	Flight Ground	7 6	mRNA and lncRNA
GSE100932	2019	Italy	GPL11154	Flight Ground	6 3	miRNA

### 2.1 GEO datasets

The relevant RNA sequencing data and microarray data were extracted from the Gene Expression Omnibus (GEO) database^1^. RNA expression profiles were derived from GSE100930 (Microarray on mRNA and lncRNA, comprising seven flight samples and six ground samples) and GSE100932 (miRNA-seq, including six flight samples, and three ground samples). These datasets were generated using the platforms GPL6244 [HuGene-1_0-st] and GPL11154 [Illumina HiSeq 2000], as initially described by Bradamante and co-workers.

### 2.2 Data preprocessing

The Bioconductor software package 3.8^2^ and the R v.3.5.2^3^ were utilized to preprocess the gene expression datasets. Specifically, the R software was employed via the Agilent platform to conduct preprocessing and normalization of the Series Matrix Files (.TXT files). Principal component analysis (PCA), a mathematical algorithm commonly integrated into genome-wide expression studies, was employed. PCA condenses data while preserving the bulk of its variability, facilitating visual assessment of sample similarities and differences and enabling grouping determination (14). In this study, PCA was conducted using the PRCOMP function in R software based on singular value decomposition (SVD) of the data matrix.

### 2.3 Identification of DEmRNAs and DEmiRNAs

The limma package in R was employed to perform differential expression analysis of microarray data between Flight and Ground conditions, focusing on mRNA expression changes. Meanwhile, for miRNAs, the edgeR package in R was utilized with RNA sequencing data. A significance threshold of *p* < 0.05 and a log2 (fold-change) > 1 were utilized as cut-off thresholds.

### 2.4 Prediction of target mRNAs for DEmiRNAs

The multiMiR package in R, comprising 14 databases, was used to predict miRNA-mRNA interactions. The predictions mainly employed data from three databases: miRecords, miRTarBase^4^ and DIANA TarBase v.8.

### 2.5 Construction of co-expressed gene module for lncRNA-mRNA pairs

Weighted gene co-expression network analysis is a state-of-the-art technique for deciphering co-expression patterns among genes (15). Firstly, from the GSE100930 dataset, 5,000 genes exhibiting the highest average expression were identified. Subsequently, the soft-thresholding power was measured, guided by the criterion of approximate scale-free topology fitting indices R^2^, whereby the β value was selected once R^2^ achieved 0.85. Employing the appropriate soft threshold (β = 12), the weighted network underwent transformation into a topological overlap matrix (TOM), utilized thereafter to compute a gene’s network connectivity. Following this, average linkage hierarchical clustering was performed using the TOM-based dissimilarity measure, enabling the classification of genes with analogous expression profiles into distinct gene modules. Ultimately, to acquire positive lncRNA-mRNA co-expression pairs from the gene modules, the TOMType parameter in the WGCNA package was designated as “signed.” The detailed code is in the Supplementary Data Sheets 1, 2.

### 2.6 Establishment of the mRNA-miRNA-lncRNA network

The positive lncRNA-mRNA co-expression pairs and miRNA-mRNA expression pairs were integrated based on shared mRNAs, with those containing DEmRNAs being filtered out. All mRNA-miRNA-lncRNA regulatory axes constituted the ceRNA network, which was visualized using Cytoscape v.3.7.2.

### 2.7 Enrichment analysis

Functional annotation of the DEmRNAs within the ceRNA network was conducted using the clusterProfiler package in R. GO terms were employed for functional enrichment analysis, while KEGG pathways were utilized for pathway enrichment analysis. A threshold of *p* < 0.05 was utilized as the cutoff for reporting significant GO terms and KEGG pathways.

### 2.8 Establishment of PPI network and identification of hub genes

The genes identified through the aforementioned analyses were cross-referenced with the STRING database^5^ to construct a PPI network. Interactions were restricted to Homo sapiens to ensure the relevance of the results to human biology. A confidence score threshold of ≥ 0.4 (medium confidence) was set to ensure that only interactions with appropriate evidence were included in the analysis. Additionally, the constructed PPI network was imported into Cytoscape software (version 3.7.2) for visualization and analysis. Finally, the CytoHubba plugin in Cytoscape software was utilized to identify the top 10 hub genes using the Maximal Clique Centrality (MCC) algorithm.

### 2.9 CMap query

The CMap dataset of cellular signatures captures the transcriptional responses of human cells to both genetic and chemical perturbations. This comprehensive dataset encompasses profiles of 27,927 perturbagens, resulting in a total of 476,251 expression signatures (16). Subsequently, the upregulated and downregulated genes were compiled into respective upregulated and downregulated signatures, accessible via https://clue.io. Using pattern-matching algorithms, connectivity scores were computed to discern functional associations among drugs, genes, and diseases based on shared gene expression changes. These scores represent the degree of similarity with the expression profile, enabling the identification of bioactive compounds with the lowest connectivity scores, which are deemed potential therapeutic drugs for treating diseases.

### 2.10 Cell model

Sprague-Dawley (SD) rat BMSCs were generously supplied by the Stem Cell Bank, Chinese Academy of Sciences. These BMSCs were cultivated in growth medium consisting of α-MEM containing 10% FBS and antibiotics (HyClone, United States). Cultures were maintained at 37°C in a 5% CO_2_ atmosphere. BMSCs assigned to the flight group were cultured in the Rotary Cell Culture System (RCCS, Synthecon Company, United States) for 72 h. During this period, the rotation speed was gradually increased from 10 to 20 (Supplementary Figures 1, 2).

### 2.11 Validation of key genes and ceRNA network by qRT-PCR

Total RNA was isolated using Eastep^®^-Super-Total-RNA-Extraction-Kit (Promega Biological Products, China). The yield of total RNA was examined using NanoDrop equipment (Thermo-Fisher-Scientific, United States). cDNA synthesis was performed using PrimeScript RT reagent Kit (Takara, Japan). Then, SYBR Green (TianGen, China) was employed to conduct qRT-PCR. All specific primer pairs (5′ to 3′) were shown as follows: (1) GAPDH: F: GGGTGTGAACCACGAGAAAT, R: ACTGTGGTCATGAGCCCTTC; (2) JPX: F: GCGAAGGTCTTG GTCACATCTGTC, R: AGAGGAGGGAAGGAAGGAAGGAAAC; (3) LINC01123: F: AGGAAGGAGGTGCTTGGCTCTC, R: TGAC AACGATGACGAGGAAACTGAC; (4) LAMC1: F: TGGACTT ACTTGCTGACTCATTGACTG, R: ATTGATGGATGGATGGAT GGATGGATG; (5) LAMA4: F: AACTGACCGAGGCTGTCAAG, R: TGAGGTTTCTCACTGCGTCC. The relative expression of genes was determined using the 2^–ΔΔCt^ approach, with GAPDH serving as the internal control.

### 2.12 Statistical analysis

The findings obtained from qRT-PCR were analyzed by Student’s *t*-test using GraphPad Prism 9.0 (GraphPad Software, Inc., United States). Statistical significance was deemed at a threshold of *P* < 0.05.

## 3 Results

### 3.1 Comparative analysis of miRNA and mRNA expression profiles in BMSCs from ground and flight samples

**FIGURE 2:**
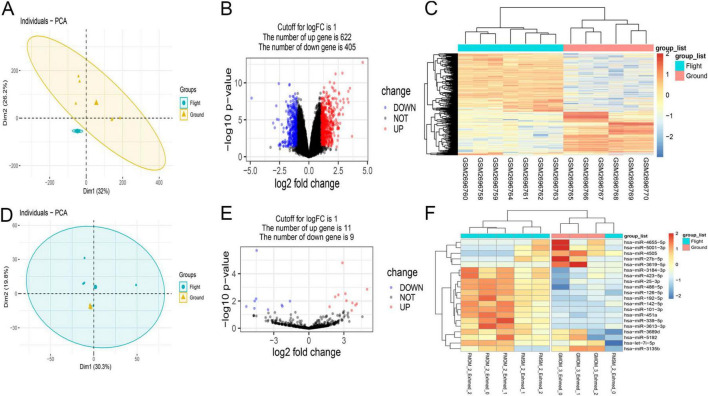
Overview of aberrantly expressed mRNAs **(A–C)** and miRNAs **(D–F)** in flight samples. **(A,D)** In principal component analysis (PCA), mRNA samples showed similarities within the group, while ground samples of miRNA did not show good similarities due to the small sample size. **(B,E)** The Volcano plot illustrating the results of mRNA and miRNA differential expression analysis. Each data point corresponds to a single mRNA or miRNA, with red indicating upregulation and blue indicating downregulation. **(C,F)** Hierarchical clustering revealed distinct mRNA or miRNA expression patterns between the flight and ground groups, demonstrating group homogeneity within each set. Each row represents a gene, and each column represents a sample. Genes with upregulation are presented as red, while those with downregulation are presented as blue, reflecting the degree of expression alteration.

In this study, 1,027 significant DEmRNAs (622 with upregulation and 405 with downregulation) were identified using a significance threshold of *p* < 0.05 and a log2-fold change of > 1 (Figures 2A–C). Additionally, 20 DEmiRNAs were discerned in BMSCs using the same criteria, comprising nine with downregulation and 11 with upregulation (Figures 2D–F). In total, 8,623 target mRNAs of the DEmiRNAs were estimated through miRecords, miRTarBase, and DIANA TarBase v.8.

### 3.2 Construction of co-expressed gene module for lncRNA-mRNA pairs

**FIGURE 3:**
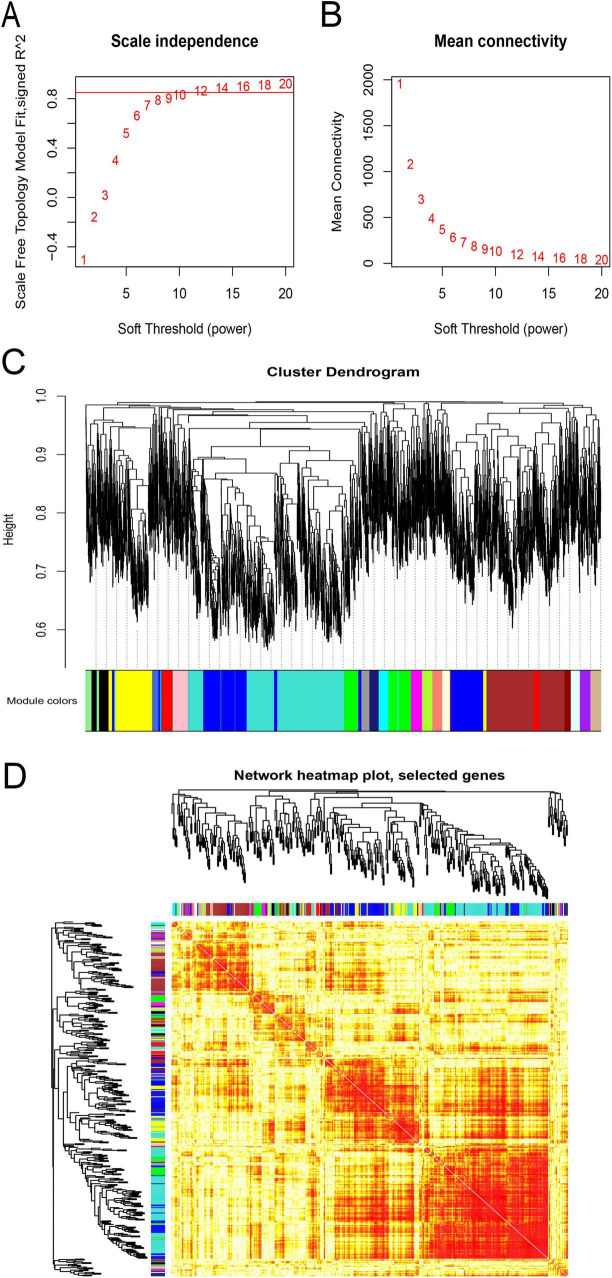
Weighted gene co-expression network analysis (WGCNA) of flight samples (GSE100930). **(A)** Assessment of scale-free fit indices across soft-thresholding powers (β). **(B)** Evaluation of mean connectivity across soft-thresholding powers. **(C)** The hierarchical clustering dendrogram was constructed according to the gene dissTOM. Each color-coded row beneath the dendrogram represents a distinct module, with module width indicating the number of co-expressed genes. **(D)** Visualization of inter-gene interactions within co-expression modules. Various colors on the vertical and horizontal axes denote diverse modules, while yellow hues denote inter-module correlations. Significant variations in correlation patterns among modules were observed.

According to the expression’s variance, the top 5,000 genes were first identified from flight samples in the GSE100930 dataset. Subsequently, through optimization, a soft threshold of β = 12 was determined, achieving an R^2^ of 0.85 (Figures 3A, B). When the TOMType parameter in the WGCNA package was set to “signed,” 21 modules were delineated via average linkage hierarchical clustering (Figures 3C, D). Ultimately, the analysis yielded 19,935 positive lncRNA-mRNA co-expression pairs across these 21 signed modules, involving 95 lncRNAs and 4,023 mRNAs.

### 3.3 Establishment of the lncRNA–miRNA–mRNA network

**FIGURE 4:**
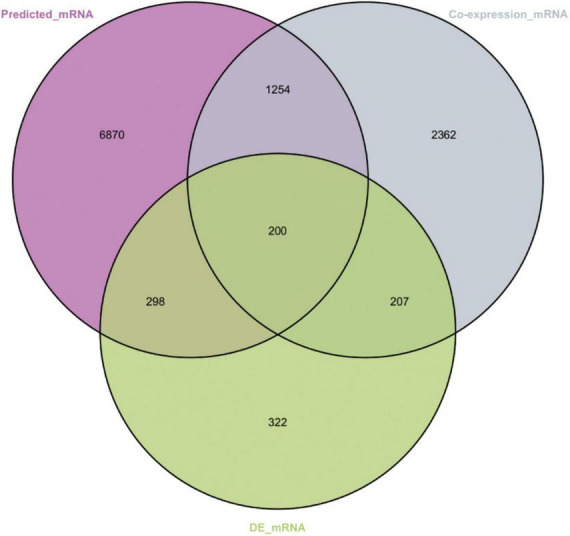
Venn diagram showing the number of shared mRNAs. The purple represents target mRNAs predicted by DEmiRNAs. The gray represents mRNAs co-expressed with long non-coding RNA (lncRNAs). The green represents differentially expressed mRNAs.

**FIGURE 5:**
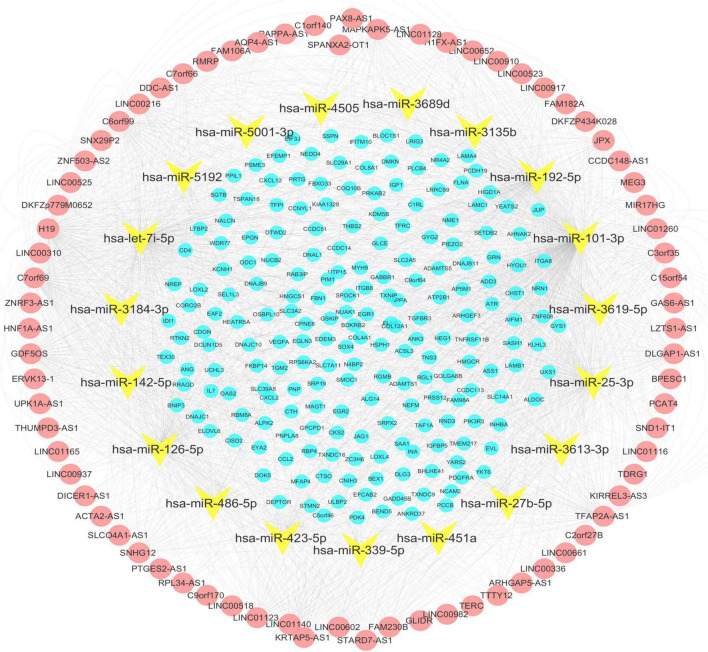
The lncRNA–miRNA–mRNA network. Nodes in yellow are miRNAs. The red nodes represent the long non-coding RNA (lncRNAs) positively correlated with the shared mRNAs. The blue nodes represent shared mRNAs. The lines represent relationships between genes.

A total of 200 shared mRNAs were obtained from positive lncRNA-mRNA co-expression pairs, predictive miRNA-mRNA expression pairs, and DEmRNAs (Figure 4). A total of 74 lncRNAs, 19 miRNAs and 200 mRNAs were then integrated into a lncRNA–miRNA–mRNA network by shared mRNAs (Figure 5), which consisted of 293 nodes and 2,075 edges.

### 3.4 Enrichment analysis

**FIGURE 6:**
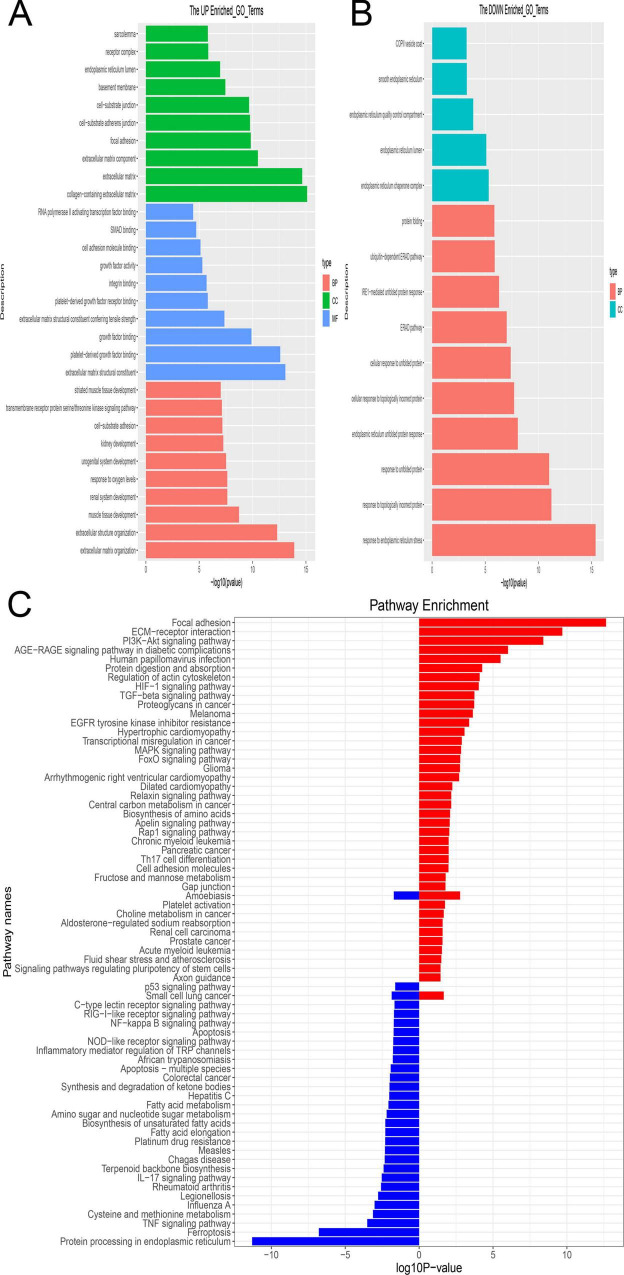
The enrichment analysis of shared mRNA. **(A,B)** GO enrichment analysis was performed across three functional categories: Molecular Functions (MF), Cellular Compartments (CC) and Biological Processes (BP). Panel **(A)** indicates enrichment by upregulated genes, while Panel **(B)** indicates enrichment by downregulated genes. The y-axis denotes the enriched GO term, while the x-axis denotes the corresponding *p*-value. **(C)** Enrichment analysis of shared mRNAs using the KEGG pathways. The enriched pathway is depicted in red, comprising upregulated genes, while the enriched pathway is shown in blue, comprising downregulated genes.

The 200 differentially expressed mRNAs (DEmRNAs) implicated in the competing endogenous RNA (ceRNA) network underwent enrichment analysis. Utilizing GO terms for Molecular Functions (MF), Cellular Compartments (CC), and Biological Processes (BP), the primary functions of these DEmRNAs were examined. Notably, significant enrichment was observed for GO terms associated with the collagen-containing extracellular matrix, extracellular matrix structural constituent, and extracellular matrix organization (Figure 6A). Conversely, enrichment analysis of downregulated genes highlighted terms associated with the endoplasmic reticulum chaperone complex and response to endoplasmic reticulum stress as the most significantly enriched (Figure 6B). These processes are likely involved in BMSC differentiation.

Moreover, KEGG pathways were employed for systematic gene characteristic analysis by integrating higher-level pathway information with gene data. Focal adhesion emerged as the most highly enriched pathway among DEmRNAs with upregulation, whereas protein processing in the endoplasmic reticulum ranked as the most enriched pathway among DEmRNAs with downregulation (Figure 6C).

### 3.5 Establishment of PPI network and identification of hub genes

**FIGURE 7:**
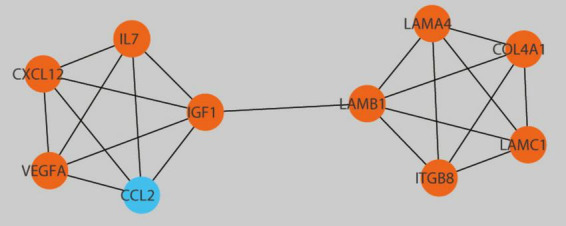
Detection of central genes within the PPI network utilizing the Cytohubba plugin in Cytoscape. Hub genes with upregulation are presented as red, while those with downregulation are presented as blue.

The selection of 10 hub genes was guided by the Cytohubba plug-in in Cytoscape. The genes, including nine upregulated genes and one downregulated gene, were then ranked according to their Maximal clique centrality (MCC) values (Figure 7). The key genes Laminin-Subunit-Gamma-1 (LAMC1) and Laminin-Subunit-Alpha-4 (LAMA4) were identified. Furthermore, from the constructed ceRNA network, three distinct mRNA-miRNA-lncRNA regulatory axes were delineated: LINC01123/hsa-let-7i-5p/LAMC1, JPX/hsa-miRNA-3619-5p/LAMA4, and LINC01123/hsa-miRNA-3619-5p/LAMA4. Detailed information regarding the two key lncRNAs, LINC01123 and JPX, is provided in Table 2.

**TABLE 2:** Basic information on the two long non-coding RNAs (lncRNAs).

LncRNA	Entrez_ID	Locus	Genomic length	Strand
JPX	554203	chrX:73,944,182-74,070,832	126651	+
LINC01123	440894	chr2:109,986,583-109,996,414	9832	+

### 3.6 CMap analysis

After submitting 200 DEmRNAs into the CMap Query, including 117 upregulated signatures and 83 downregulated signatures, three bioactive compounds (SB-216763, oxymetholone, flubendazole) were identified as candidate therapeutics for treating DOP since they had the lowest connectivity scores (Table 3).

**TABLE 3:** Three compounds with high negative connectivity scores in Connectivity Map (CMap) analysis.

Name	ID	Target	Score	MOA
SB-216763	BRD-K59184148	GSK3B, CCNA2, CDK2, GSK3A	−97.86	Glycogen synthase kinase inhibitor
Oxymetholone	BRD-A23637604	AR	−97.55	Androgen receptor agonist
Flubendazole	BRD-K86003836	TUBB	−96.54	Tubulin inhibitor

### 3.7 Validation of key genes and ceRNA network in DOP

**FIGURE 8:**
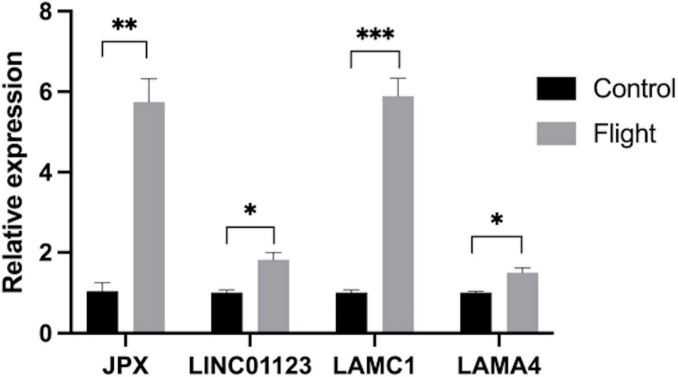
Expression of JPX, LINC01123, LAMC1 and LAMA4 in samples from rats in the flight group (in gray) and the control group (in black) (*P < 0.05, **P < 0.01,***P < 0.001).

To validate the findings from the microarray analysis, qRT-PCR was employed to detect the expression levels of key genes and the ceRNA network (Figure 8). The microarray analysis indicated upregulation of LAMC1 and LAMA4 in the DOP condition. According to the competitive mechanism, their corresponding lncRNAs were expected to exhibit upregulation in DOP as well. The qRT-PCR results demonstrated high expression levels of JPX, LINC01123, LAMC1, and LAMA4 in the flight group, aligning with our anticipated outcomes.

## 4 Discussion

Disuse osteoporosis is a prevalent condition experienced by both astronauts during spaceflight and individuals with spinal cord injuries, contributing significantly to deleterious effects on human health (5, 17). Is widely recognized that a majority of human RNA transcripts do not encode proteins but rather play crucial roles in regulating cellular physiology and influencing cellular function. Recent advances in network biology have highlighted the importance of certain properties of biological networks (18), such as scale-free distribution and hierarchical modularization, in maintaining the stability of cellular processes. This underscores the potential of regulatory network analysis to offer novel insights into the mechanisms underlying gene regulation and dysfunction in DOP. In this study, we present, for the first time, the construction of a ceRNA network for BMSCs in DOP, linking the functions of protein-coding mRNAs with non-coding RNAs, including lncRNAs and miRNAs.

Through functional enrichment analysis, we propose a hypothesis that following the cessation of mechanical stress, dysregulated lncRNAs may disrupt signaling pathways involved in endoplasmic reticulum (ER) stress and extracellular matrix (ECM) collagen production. This disruption, in turn, could impact bone mineral deposition, turnover, and osteocyte activity (19). ER stress, associated with cellular damage to the protein-folding mechanism, has been reported to inhibit osteoblast formation, increase osteoclast numbers, and enhance their migration and adhesion, consistent with our enrichment analysis findings (20). Notably, focal adhesion emerged as a significant term in both GO and KEGG analyses. Focal adhesion plays a crucial role in mediating cellular interactions with the ECM and transducing mechanical forces and regulatory signals (21). Furthermore, in vivo studies have demonstrated the assembly of focal adhesion complexes in response to mechanical overload, suggesting its potential involvement in DOP development (22, 23). However, further investigation is warranted to validate this observation. This underscores the importance of understanding the effects of microgravity on osteoblast cytoskeletal adhesion and organization as well as osteoclast bone resorption in regulating DOP progression.

The PPI network analysis revealed two hub genes, LAMC1 and LAMA4. LAMC1, a pivotal member of the laminin family, interacts with integrins, promoting cell migration and adhesion (24). Additionally, LAMC1 plays a significant role in aging BMSCs (25). LAMA4, a key component of the bone marrow ECM, influences the proliferation and survival of stem and progenitor cells (26). Both hub genes are associated with the ECM term (GO: 0031012) in GO analysis. The mineralization process of bone tissue exhibits unique biomechanical properties, and the investigation into vertebrate extracellular matrix mineralization remains a focal point in research (27). Bone comprises hundreds of ECM proteins, and the ECM of various bone tissue compartments directs bone remodeling through the coordinated actions of osteoblasts and osteoclasts (28). Exploring key genes within the ECM could elucidate its role in DOP.

In our study, two key regulatory axes, JPX/hsa-miR-3619-5p/LAMA4 and LINC01123/hsa-let-7i-5p/LAMC1, were identified as critical modulators of ECM remodeling and bone homeostasis. Both JPX and LINC01123 function as competitive endogenous RNAs (ceRNAs), sequestering miRNAs to prevent the suppression of LAMA4 and LAMC1, two essential ECM components. This enhances ECM structural stability, promotes osteoblast attachment and proliferation, and modulates osteoclast activity, thereby influencing bone metabolism.3.

The lncRNA JPX, known as an activator of Xist, can activate Xist both in trans and cis (29). XIST has been implicated in promoting osteoporosis by inhibiting the differentiation of BMSCs (30, 31). Moreover, the Wnt/β-catenin pathway, crucial for DOP development, may be activated by hsa-miR-3619-5p through enhancing the stability of β-catenin (32). Our findings suggest that JPX, acting as a ceRNA, modulates hsa-miR-3619-5p levels, influencing the expression of LAMA4 and the remodeling of ECM. Dysregulation of this axis could destabilize bone matrix integrity and disrupt osteoblast and osteoclast activity. Activation of Wnt signaling via hsa-miR-3619-5p may partially mitigate bone loss associated with DOP, presenting a potential therapeutic strategy. The JPX/hsa-miR-3619-5p/LAMA4 axis thus emerges as a critical regulatory mechanism in ECM remodeling and DOP progresion.

Previous studies have demonstrated the ceRNA crosstalk network involving LINC01123, which acts as a decoy to sequester miRNA-199a-5p from binding c-Myc mRNA, thereby attenuating its suppressive effect on c-Myc expression (33). Although hsa-let-7i-5p has not been extensively studied in bone-related research, it has shown potential as a diagnostic and predictive biomarker in other disease models (34, 35). Further investigation is warranted to elucidate the potential functions of these lncRNAs and miRNAs. In our study, LINC01123 was identified as a competitive endogenous RNA (ceRNA) that sequesters hsa-let-7i-5p, thereby upregulating LAMC1 expression and supporting ECM integrity. Dysregulation of this axis, particularly the overexpression of LINC01123, could impair osteoblast differentiation and enhance osteoclast activity, disrupting bone matrix mineralization and turnover. This dysregulation may exacerbate the pathological progression of disuse osteoporosis (DOP), highlighting the critical role of the LINC01123/hsa-let-7i-5p/LAMC1 axis in maintaining bone homeostasis under mechanical unloading conditions.

Based on CMap analysis, three chemicals, namely, SB-216763, Oxymetholone, and Flubendazole, emerged as potential therapeutic agent for treating DOP. SB-216763, an inhibitor of glycogen synthase kinase 3 (GSK-3), regulates osteoclast differentiation by phosphorylating NFATc1. Moreover, GSK-3, activated by dexamethasone, inhibits the osteoblast differentiation-related cell cycle (36–38). Notably, GSK-3 also plays a crucial role in the Wnt/β-catenin axis, a potential target for osteoporosis treatment (39, 40). Therefore, SB-216763 holds promise as a significant drug candidate for DOP treatment.

Oxymetholone is an androgen and anabolic steroid (AAS) which is used for treating osteoporosis (41). Its anabolic effects are mediated through androgen receptor activation, which stimulates protein synthesis and mineral deposition in bone tissue. Oxymetholone has a well-established clinical history in treating osteoporosis and other conditions associated with bone fragility. Its ability to enhance bone mass and strength makes it a valuable option for managing severe DOP, particularly in patients with significant bone loss due to prolonged immobilization. Our data were consistent with previous results; and to avoid repetition of previously published discussion, the data will not be examined in depth here.

Flubendazole, a tubulin inhibitor, has been extensively evaluated in humans and animals for the treatment of intestinal parasites and systemic worm infections. Tubulin is a major component of microtubules, which are cytoskeletal components needed for cell division, cell transport, and cell integrity (42). The microtubule-disrupting mechanism underpinning flubendazole’s antiproliferative effects has garnered significant interest in oncology research. Preclinical studies have demonstrated its broad-spectrum anti-tumor efficacy across diverse malignancies (43, 44). In addition, previous studies showed that another tubulin inhibitor vinpocetine had a similar binding domain like that for flubendazole, and was shown to inhibit RANKL-induced osteoclastogenesis and thereby attenuate bone loss (45). However, the direct effects of flubendazole on bone metabolism, particularly concerning osteoblasts and osteoclasts, remain inadequately explored. As a benzimidazole anthelmintic agent, flubendazole demonstrates a well-established safety profile at standard therapeutic doses, with minimal incidence of severe adverse reactions in clinical practice (46, 47). However, its pharmacological safety requires rigorous reassessment when considering potential applications in bone disorders, particularly in the context of bone-related pathologies where drug-bone cell interactions remain undefined. That flubendazole may be a potential drug for reducing bone loss after DOP is also worthy of further study.

However, we acknowledge several limitations in this research. Firstly, the sample size of BMSCs in the dataset was small, although it met the statistical significance requirements for biological duplication. The relatively small sample size may limit the robustness of miRNA differential expression analysis and we will integrate larger datasets in future research to enhance the reliability of the results. Secondly, although we constructed the ceRNA network, further investigation is needed to elucidate the precise mechanisms of action of these lncRNAs, miRNAs, and mRNAs through targeted studies. Integrating more data in the future will enhance the accuracy and completeness of the ceRNA network. As our future direction, we aim to explore the direct molecular biological mechanisms of DOP-specific ceRNAs and how dysregulated ECM remodeling contributes to DOP. Additionally, further studies on bioactive compounds will investigate their potential in translational medicine.

## 5 Conclusion

In conclusion, through bioinformatics analysis of BMSC gene expression data under microgravity conditions, we constructed the first mRNA-miRNA-lncRNA network. This network sheds light on the potential role of ceRNA in regulating BMSCs under microgravity and provides novel insights into the regulatory mechanisms and therapeutic targets for DOP.

## Data Availability

The original contributions presented in this study are included in this article/Supplementary material, further inquiries can be directed to the corresponding author.

## Footnotes

1
http://www.ncbi.nlm.nih.gov/geo/

2
https://www.bioconductor.org/

3
https://www.r-project.org

4
http://mirtarbase.mbc.nctu.edu.tw/php/index.php

5
https://string-db.org/
