# Supplementary figures and images for: Deciphering the role of lncRNA-mediated ceRNA network in disuse osteoporosis: insights from bone marrow mesenchymal stem cells under simulated microgravity
Source: Front Med (Lausanne). 2025 Apr 3;12:1444165. doi: 10.3389/fmed.2025.1444165 (PMC12003301; doi:10.3389/fmed.2025.1444165)

Module-trait relationships

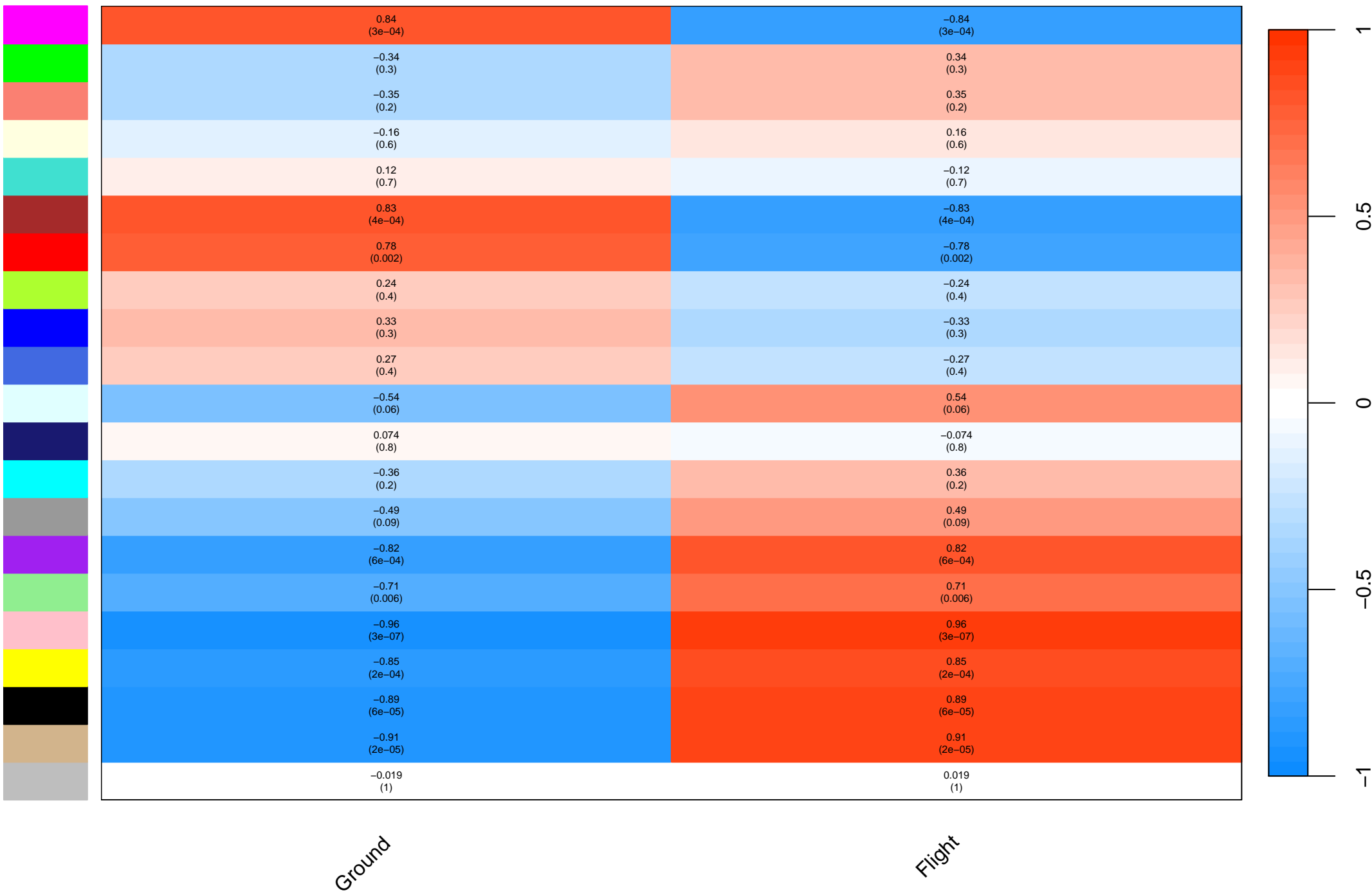

Supplement: Supplementary file 1 [file Data_Sheet_1.pdf]

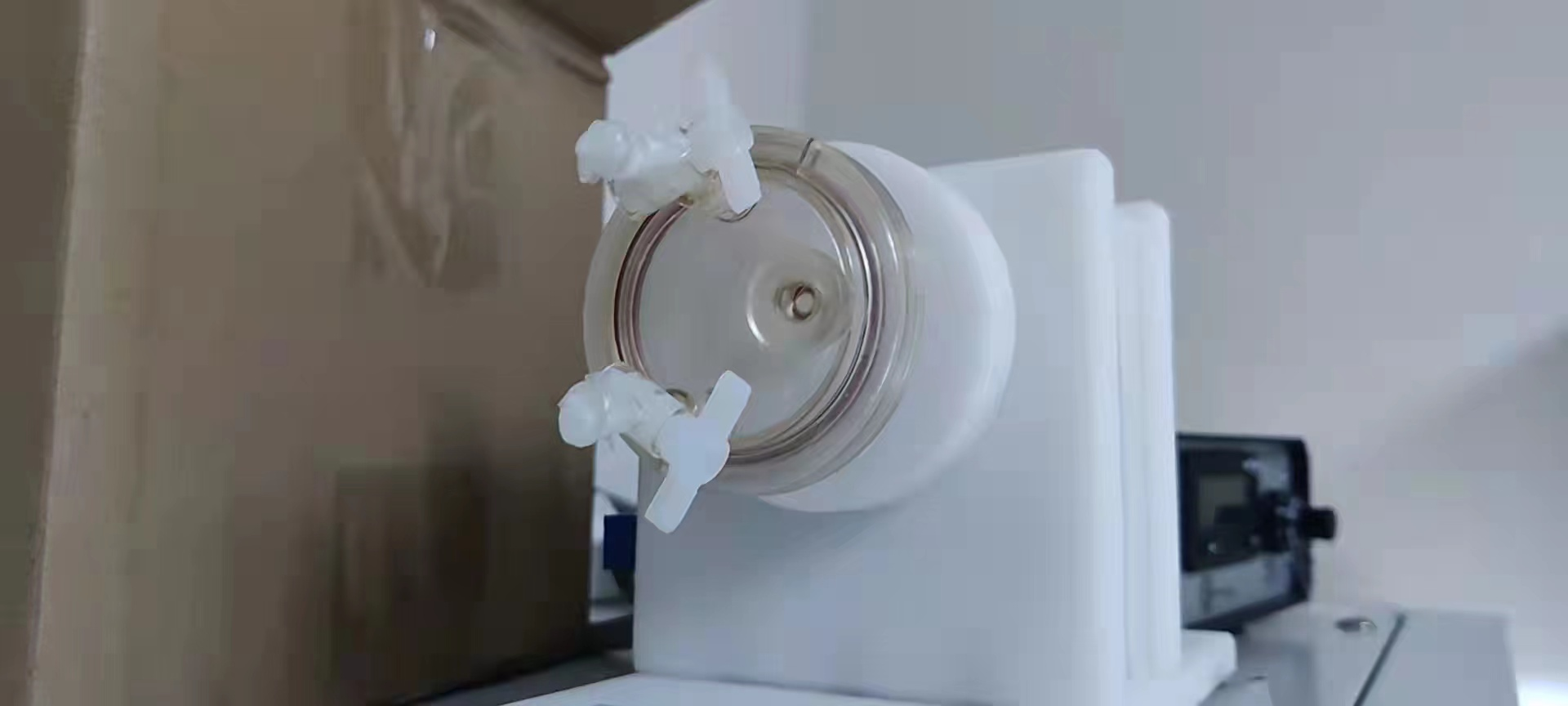

Supplement: Supplementary file 3 [file Image_1.tiff]

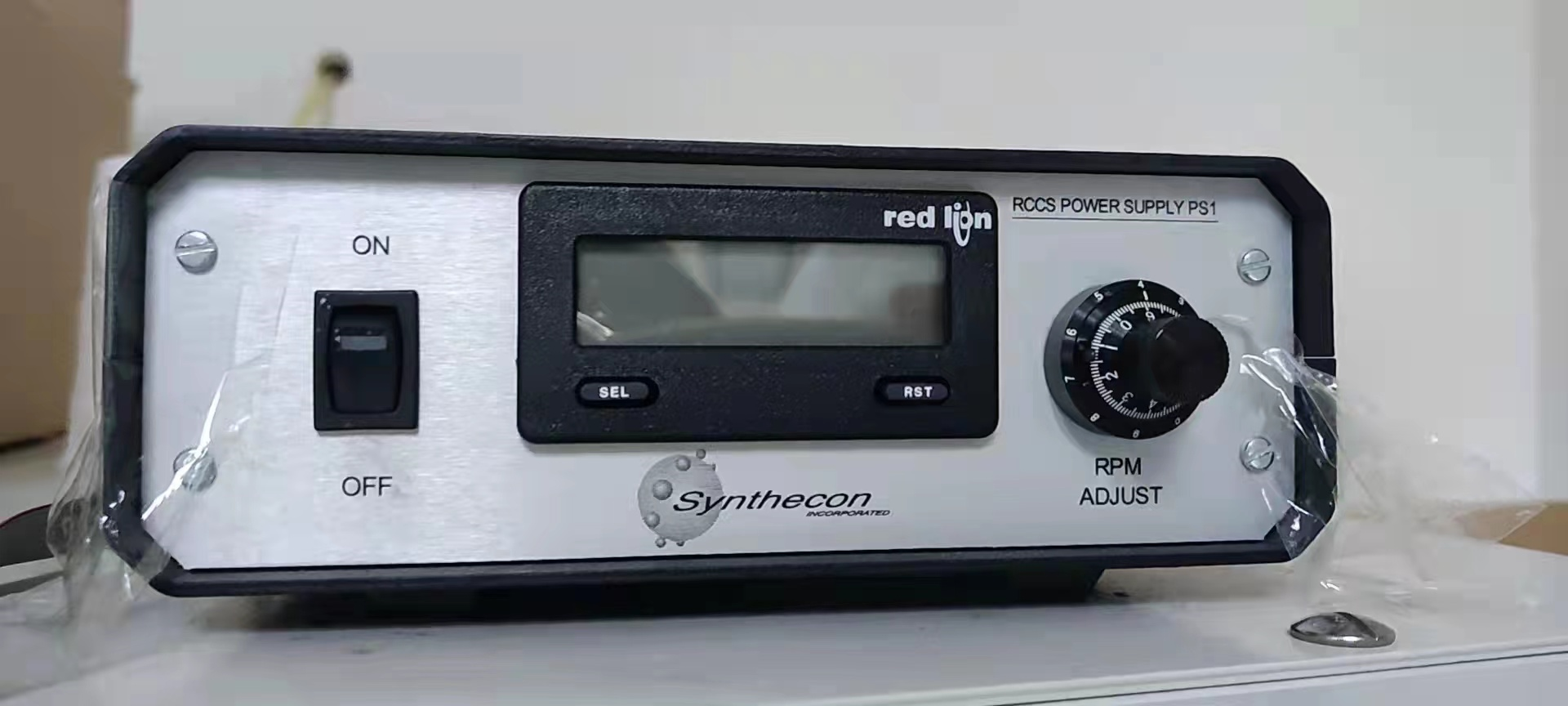

Supplement: Supplementary file 4 [file Image_2.tiff]
