# Supplementary material for: Deciphering the role of lncRNA-mediated ceRNA network in disuse osteoporosis: insights from bone marrow mesenchymal stem cells under simulated microgravity
Source: Front Med (Lausanne). 2025 Apr 3;12:1444165. doi: 10.3389/fmed.2025.1444165 (PMC12003301; doi:10.3389/fmed.2025.1444165)
Supplement: Supplementary file 2 [file Data_Sheet_2.pdf]

```
rm(list = ls())
options(stringsAsFactors = F)
load('GSE100930_normalize.Rdata')
  metadata=phe
  datTraits = data.frame(gsm=metadata[,2],

cellline=trimws(sapply(as.character(metadata$characteristics_ch1.1),
function(x) strsplit(x,":")[[1]][2])),

subtype=trimws(sapply(as.character(metadata$characteristics_ch1.2),f
unction(x) strsplit(x,":")[[1]][2]))
             )
  save(exprSet,datTraits,file = 'GSE100930-wgcna-input.RData')
library(WGCNA)
## step 1 :
if(T){
  fpkm<-exprSet

  fpkm[1:4,1:4]
  head(datTraits)
  table(datTraits$cellline)
  RNAseq_voom <- fpkm

  WGCNA_matrix = t(RNAseq_voom[order(apply(RNAseq_voom,1,mad),
decreasing = T)[1:5000],])
  datExpr0 <- WGCNA_matrix  ## top 5000 mad genes
  datExpr <- datExpr0

  sampleNames = rownames(datExpr);
  traitRows = match(sampleNames, datTraits$gsm)
  rownames(datTraits) = datTraits[, 1]

}


## step 2
datExpr[1:4,1:4]
if(T){
  powers = c(c(1:10), seq(from = 12, to=20, by=2))
  # Call the network topology analysis function
  sft = pickSoftThreshold(datExpr, powerVector = powers, verbose =
5)
  png("step2-beta-value.png",width = 800,height = 600)
  # Plot the results:
  ##sizeGrWindow(9, 5)
  par(mfrow = c(1,2));
  cex1 = 0.9;
  # Scale-free topology fit index as a function of the soft-
thresholding power
  plot(sft$fitIndices[,1],
-sign(sft$fitIndices[,3])*sft$fitIndices[,2],
       xlab="Soft Threshold (power)",ylab="Scale Free Topology Model
Fit,signed R^2",type="n",
       main = paste("Scale independence"));
```

```
  text(sft$fitIndices[,1],
-sign(sft$fitIndices[,3])*sft$fitIndices[,2],
       labels=powers,cex=cex1,col="red");
  # this line corresponds to using an R^2 cut-off of h
  abline(h=0.70,col="red")
  # Mean connectivity as a function of the soft-thresholding power
  plot(sft$fitIndices[,1], sft$fitIndices[,5],
       xlab="Soft Threshold (power)",ylab="Mean Connectivity",
type="n",
       main = paste("Mean connectivity"))
  text(sft$fitIndices[,1], sft$fitIndices[,5], labels=powers,
cex=cex1,col="red")
  dev.off()
}

## step 3
if(T){
  net = blockwiseModules(
    datExpr,
    power = 12,
    maxBlockSize = 6000,
    TOMType = "signed", minModuleSize = 20,
    reassignThreshold = 0, mergeCutHeight = 0.15,
    numericLabels = TRUE, pamRespectsDendro = FALSE,
    saveTOMs = F,
    verbose = 3
  )
  table(net$colors)
}
## step 4
if(T){

  # Convert labels to colors for plotting
  mergedColors = labels2colors(net$colors)
  table(mergedColors)
  moduleColors=mergedColors
  # Plot the dendrogram and the module colors underneath
  png("step4-genes-modules.pdf",width = 8,height = 6)
  plotDendroAndColors(net$dendrograms[[1]],
mergedColors[net$blockGenes[[1]]],
                      "Module colors",
                      dendroLabels = F, hang = 0.03,
                      addGuide = TRUE, guideHang = 0.05)
  dev.off()
  ## assign all of the gene to their corresponding module
  ## hclust for the genes.
}
gene_module <- data.frame(ID=colnames(datExpr), module=moduleColors)
gene_module = gene_module[order(gene_module$module),]
write.table(gene_module,file='gene_module',
            sep='\t',quote=F,row.names=F)
if(F){
  nGenes = ncol(datExpr)
  nSamples = nrow(datExpr)
```

```
  datExpr_tree<-hclust(dist(datExpr), method = "average")
  par(mar = c(0,5,2,0))
  plot(datExpr_tree, main = "Sample clustering", sub="", xlab="",
cex.lab = 2,
       cex.axis = 1, cex.main = 1,cex.lab=1)
  sample_colors <-
numbers2colors(as.numeric(factor(datTraits$subtype)),
                                  colors =
c("blue","white","red","green"),signed = FALSE)
  #  sample_colors <- numbers2colors( datTraits ,signed = FALSE)
  par(mar = c(1,4,3,1),cex=0.8)

  png("sample-subtype-cluster.png",width = 800,height = 600)
  plotDendroAndColors(datExpr_tree, sample_colors,
                      groupLabels = colnames(sample),
                      cex.dendroLabels = 0.8,
                      marAll = c(1, 4, 3, 1),
                      cex.rowText = 0.01,
                      main = "Sample dendrogram and trait heatmap")
  dev.off()
}

## step 5
table(datTraits$cellline)
if(T){
  nGenes = ncol(datExpr)
  nSamples = nrow(datExpr)
  design=model.matrix(~0+datTraits$cellline)
  design2=model.matrix(~0+datTraits$subtype)
  design<-cbind(design,design2)
  colnames(design)=levels(datTraits$cellline)
  colnames(design)=c('Ground','Flight')
  moduleColors <- labels2colors(net$colors)
  # Recalculate MEs with color labels
  MEs0 = moduleEigengenes(datExpr, moduleColors)$eigengenes
  MEs = orderMEs(MEs0);
  moduleTraitCor = cor(MEs, design , use = "p");
  moduleTraitPvalue = corPvalueStudent(moduleTraitCor, nSamples)

  sizeGrWindow(10,6)
  # Will display correlations and their p-values
  textMatrix = paste(signif(moduleTraitCor, 2), "\n(",
                     signif(moduleTraitPvalue, 1), ")", sep = "");
  dim(textMatrix) = dim(moduleTraitCor)
  png("newstep5-Module-trait-relationships.png",width = 800,height =
1200,res = 120)
  par(mar = c(6, 8.5, 3, 3));
  # Display the correlation values within a heatmap plot
  labeledHeatmap(Matrix = moduleTraitCor,
                 xLabels = colnames(design),
                 yLabels = names(MEs),
                 ySymbols = names(MEs),
                 colorLabels = FALSE,
                 colors = blueWhiteRed(50),
```

```
                 textMatrix = textMatrix,
                 setStdMargins = FALSE,
                 cex.text = 0.5,
                 zlim = c(-1,1),
                 main = paste("Module-trait relationships"))
  dev.off()

  flight = as.data.frame(design[,2]);
  names(flight) = "flight"
  y=flight
  GS1=as.numeric(cor(y,datExpr, use="p"))
  GeneSignificance=abs(GS1)
  # Next module significance is defined as average gene
significance.
  ModuleSignificance=tapply(GeneSignificance,
                            moduleColors, mean, na.rm=T)
  sizeGrWindow(8,7)
  par(mfrow = c(1,1))

  plotModuleSignificance(GeneSignificance,moduleColors)

}


## step 6
Flight = as.data.frame(design[,2]);
names(Flight) = "Flight"
module = "blue"
if(T){
  # names (colors) of the modules
  modNames = substring(names(MEs), 3)
  geneModuleMembership = as.data.frame(cor(datExpr, MEs, use =
"p"));

  MMPvalue =
as.data.frame(corPvalueStudent(as.matrix(geneModuleMembership),
nSamples));
  names(geneModuleMembership) = paste("MM", modNames, sep="");
  names(MMPvalue) = paste("p.MM", modNames, sep="");
  geneModuleMembership[1:4,1:4]



  Flight = as.data.frame(design[,2]);
  names(Flight) = "Flight"
  geneTraitSignificance = as.data.frame(cor(datExpr, Flight, use =
"p"));
  GSPvalue =
as.data.frame(corPvalueStudent(as.matrix(geneTraitSignificance),
nSamples));
  names(geneTraitSignificance) = paste("GS.", names(Flight),
sep="");
  names(GSPvalue) = paste("p.GS.", names(Flight), sep="");
```

```
  module = "blue"
  column = match(module, modNames);
  moduleGenes = moduleColors==module;
  png("step6-Module_membership-gene_significance.png",width =
800,height = 600)
  #sizeGrWindow(7, 7);
  par(mfrow = c(1,1));
  verboseScatterplot(abs(geneModuleMembership[moduleGenes, column]),
                     abs(geneTraitSignificance[moduleGenes, 1]),
                     xlab = paste("Module Membership in", module,
"module"),
                     ylab = "Gene significance for flight",
                     main = paste("Module membership vs. gene
significance\n"),
                     cex.main = 1.2, cex.lab = 1.2, cex.axis = 1.2,
col = module)
  dev.off()

}


## step 7

if(T){
  nGenes = ncol(datExpr)
  nSamples = nrow(datExpr)
  geneTree = net$dendrograms[[1]];
  dissTOM = 1-TOMsimilarityFromExpr(datExpr, power = 16);
  plotTOM = dissTOM^7;
  diag(plotTOM) = NA;
  #TOMplot(plotTOM, geneTree, moduleColors, main = "Network heatmap
plot, all genes")
  nSelect = 400
  # For reproducibility, we set the random seed
  set.seed(10);
  select = sample(nGenes, size = nSelect);
  selectTOM = dissTOM[select, select];
  # There’s no simple way of restricting a clustering tree to a
subset of genes, so we must re-cluster.
  selectTree = hclust(as.dist(selectTOM), method = "average")
  selectColors = moduleColors[select];
  # Open a graphical window
  sizeGrWindow(9,9)
  # Taking the dissimilarity to a power, say 10, makes the plot more
informative by effectively changing
  # the color palette; setting the diagonal to NA also improves the
clarity of the plot
  plotDiss = selectTOM^7;
  diag(plotDiss) = NA;

  png("step7-Network-heatmap.png",width = 800,height = 600)
  TOMplot(plotDiss, selectTree, selectColors, main = "Network
heatmap plot, selected genes")
  dev.off()
```

```
  # Recalculate module eigengenes
  MEs = moduleEigengenes(datExpr, moduleColors)$eigengenes

  Luminal = as.data.frame(design[,3]);
  names(Luminal) = "Luminal"
  # Add the weight to existing module eigengenes
  MET = orderMEs(cbind(MEs, Flight))
  MET = orderMEs(MEs)

  # Plot the relationships among the eigengenes and the trait
  sizeGrWindow(5,7.5);

  par(cex = 0.9)
  png("step7-Eigengene-dendrogram.pdf",width = 800,height = 600)
  plotEigengeneNetworks(MET, "", marDendro = c(0,4,1,2), marHeatmap
= c(3,4,1,2), cex.lab = 0.8, xLabelsAngle
                        = 90)
  dev.off()

  # Plot the dendrogram
  sizeGrWindow(6,6);
  par(cex = 1.0)
  png("step7-Eigengene-dendrogram-hclust.png",width = 800,height =
600)
  plotEigengeneNetworks(MET, "Eigengene dendrogram", marDendro =
c(0,4,2,0),
                        plotHeatmaps = FALSE)
  dev.off()
  # Plot the heatmap matrix (note: this plot will overwrite the
dendrogram plot)
  par(cex = 1.0)


  png("step7-Eigengene-adjacency-heatmap.png",width = 800,height =
600)
  plotEigengeneNetworks(MET, "Eigengene adjacency heatmap",
marHeatmap = c(3,4,2,2),
                        plotDendrograms = FALSE, xLabelsAngle = 90)
  dev.off()

}

## step 8
if(T){
  # Select module
  module = "yellow";
  # Select module probes
  probes = colnames(datExpr)
  inModule = (moduleColors==module);
  modProbes = probes[inModule];
  head(modProbes)

}
```

```
## step 9
save(datExpr,sft,geneModuleMembership,GS_Flight,GS_Osteogenic,file =
'WGCNA_person.Rdata')
if(T){
  # Recalculate topological overlap
  TOM = TOMsimilarityFromExpr(datExpr, power = 16);
  # Select module
  module = 'turquoise';
  # Select module probes
  probes = colnames(datExpr)
  inModule = (moduleColors==module);
  modProbes = probes[inModule];
  # Select the corresponding Topological Overlap
  modTOM = TOM[inModule, inModule];
  dimnames(modTOM) = list(modProbes, modProbes)
  cyt = exportNetworkToCytoscape(
    modTOM,
    edgeFile = paste("CytoscapeInput-edges-", paste(module,
collapse="-"), ".txt", sep=""),
    nodeFile = paste("CytoscapeInput-nodes-", paste(module,
collapse="-"), ".txt", sep=""),
    weighted = TRUE,
    threshold = 0.02,
    nodeNames = modProbes,
    nodeAttr = moduleColors[inModule]
  );
}
```
